# Assessing clinical and biomarker characteristics to optimize the benefits of sacubitril/valsartan in heart failure

**DOI:** 10.3389/fcvm.2022.1058998

**Published:** 2022-12-22

**Authors:** Sheldon E. Litwin, Cara A. East

**Affiliations:** ^1^Division of Cardiology, Medical University of South Carolina, Charleston, SC, United States; ^2^Ralph H. Johnson Veterans Affairs Health Network, Charleston, SC, United States; ^3^Baylor Soltero Cardiovascular Research Center, Baylor Scott and White Research Institute, Dallas, TX, United States

**Keywords:** angiotensin receptor-neprilysin inhibitor, sacubitril/valsartan, heart failure, left ventricular ejection fraction, cardiac remodeling, angiotensin-converting enzyme inhibitors

## Abstract

Of the various medical therapies for heart failure (HF), sacubitril/valsartan is a first-in-class angiotensin receptor-neprilysin inhibitor that combines sacubitril, a pro-drug that is further metabolized to the neprilysin inhibitor sacubitrilat, and the angiotensin II type 1 receptor blocker valsartan. Inhibition of neprilysin and blockade of the angiotensin II type 1 receptor with sacubitril/valsartan increases vasoactive peptide levels, increasing vasodilation, natriuresis, and diuresis. Left ventricular ejection fraction (LVEF) is widely used to classify HF, to assist with clinical decision-making, for patient selection in HF clinical trials, and to optimize the benefits of sacubitril/valsartan in HF. However, as HF is a complex syndrome that occurs on a continuum of overlapping and changing phenotypes, patient classification based solely on LVEF becomes problematic. LVEF measurement can be imprecise, have low reproducibility, and often changes over time. LVEF may not accurately reflect inherent disease heterogeneity and complexity, and the addition of alternate criteria to LVEF may improve phenotyping of HF and help guide treatment choices. Sacubitril/valsartan may work, in part, by mechanisms that are not directly related to the LVEF. For example, this drug may exert antifibrotic and neurohumoral modulatory effects through inhibition or activation of several signaling pathways. In this review, we discuss markers of cardiac remodeling, fibrosis, systemic inflammation; activation of neurohormonal pathways, including the natriuretic system and the sympathetic nervous system; the presence of comorbidities; patient characteristics; hemodynamics; and HF signs and symptoms that may all be used to (1) better understand the mechanisms of action of sacubitril/valsartan and (2) help to identify subsets of patients who might benefit from treatment, regardless of LVEF.

## Highlights

- Sacubitril/valsartan benefits are most evident in patients with LVEF below normal.
- Use of LVEF to guide treatment choice in heart failure has limitations.
- Markers of cardiac remodeling and fibrosis might be useful in identifying groups with greater benefit from treatment.

## Introduction

Sacubitril/valsartan is a first-in-class angiotensin receptor-neprilysin inhibitor that combines sacubitril and valsartan, an angiotensin II type 1 receptor inhibitor (1, 2). Neprilysin is a membrane-bound peptidase that catalyzes the degradation of various endogenous peptides such as atrial natriuretic peptide (ANP), brain natriuretic peptide (BNP), and C-type natriuretic peptide (NP), as well as angiotensin II, bradykinin, substance P, and adrenomedullin (3, 4). ANP and BNP are secreted in response to myocardial or atrial stretch, which is affected by left ventricular (LV) size and volume, and function *via* the cyclic guanosine monophosphate (cGMP) pathway in target cells to stimulate vasodilation, renal excretion of sodium and water, inhibition of the renin-angiotensin-aldosterone system, and inhibition of fibrosis (3). By inhibiting neprilysin, sacubitril/valsartan prevents NP degradation, increasing ANP and BNP concentrations, which in turn increase vasodilation, natriuresis, and diuresis. In contrast, N-terminal pro B-type natriuretic peptide (NT-proBNP), which is released with BNP after cleavage of pro-BNP, is not a substrate of neprilysin and may accurately reflect the changes in myocardial wall stress following treatment (5).

Left ventricular ejection fraction (LVEF) is widely used to define different phenotypes of heart failure (HF) and responsiveness to sacubitril/valsartan and, accordingly, is a standard patient selection factor in most HF clinical trials. The normal mean value (± SD) of LVEF is 64% (± 5%; 2SD range 54–74%) in women and 62% (± 5%; 2SD range 52–72%) in men (6). A recently proposed revised classification of HF based on LVEF has the following categories: HF with reduced ejection fraction (HFrEF; LVEF ≤ 40%); HF with preserved ejection fraction (HFpEF; LVEF ≥ 50%); HF with mildly reduced ejection fraction (LVEF 41–49%) (previously called mid-range ejection fraction; HFmrEF); and HF with improved ejection fraction (baseline LVEF ≤ 40%, increase of ≥10 points from baseline and second LVEF >40%, HFimpEF) (7).

A patient’s neurohormonal and clinical characteristics may allow for more aggressive management of HF as medical therapies directed at attenuating neurohormonal activation such as sacubitril/valsartan (8) have been effective in reducing morbidity and mortality in patients with HFrEF (9, 10) but less successful in patients with HFpEF (11–13). As HFpEF is the most commonly diagnosed form of HF (13) and HFpEF diagnoses are increasing (11), several prospective, randomized, placebo-controlled clinical trials evaluating HFpEF medical therapies have been conducted or are ongoing (Table 1; 14). Since patients with HFpEF have very heterogeneous characteristics, it would be desirable to identify markers, aside from LVEF, that could potentially predict responsiveness to the therapies.

**TABLE 1:** Key clinical trials of medical therapies for heart failure with preserved ejection fraction (HFpEF) (14).

Study name	Study name abbreviation	ClinicalTrials.gov identifier
Prospective comparison of ARNI with ARB global outcomes in HF with preserved ejection fraction	PARAGON-HF	NCT01920711
Candesartan in heart failure: assessment of reduction in mortality and morbidity	CHARM-Preserved	NCT00634712
Irbesartan in heart failure with preserved systolic function	I-PRESERVE	NCT00095238
Treatment of preserved cardiac function heart failure with an aldosterone antagonist	TOPCAT	NCT00094302
Empagliflozin outcome trial in patients with chronic heart failure with preserved ejection fraction	EMPEROR-Preserved	NCT03057951
Dapagliflozin evaluation to improve the LIVEs of patients with preserved ejection fraction heart failure	DELIVER	NCT03619213

ARB, angiotensin II receptor blocker; ARNI, angiotensin receptor-neprilysin inhibitor; HF, heart failure; HFpEF, heart failure with preserved ejection fraction.

Heart failure is a complex syndrome that occurs on a continuum of overlapping and changing phenotypes, making patient classification based solely on LVEF problematic. Although LVEF is a useful parameter, it has the major limitations of relatively high inter-observer and test-retest variability (15). As LVEF does not fully reflect the pathophysiology of HF, validated markers associated with disease pathophysiology could help optimize treatment strategies for patients with HF (16). Herein we discuss some of the molecular mechanisms contributing to HF and the associated rationales for investigating alternate markers of disease states and responsiveness to sacubitril/valsartan across the spectrum of LVEF. These include serum biomarkers such as NP levels, markers of collagen and matrix activity or turnover, markers of inflammation, markers of autonomic activation; cardiac factors including pattern of cardiac remodeling, size and function of other chambers (e.g., the left atrium and right ventricle), presence of valvular disease, stroke volume and cardiac output, pulmonary artery pressures, and vascular resistance; the presence of comorbidities such as diabetes, chronic kidney disease, hypertension, liver disease, and sleep-disordered breathing; patient characteristics such as age, gender, race, and body mass index; physiological characteristics including heart rate and blood pressure; cause of HF (ischemic vs. non-ischemic); hemodynamics; and HF signs and symptoms.

## Sacubitril/valsartan for HF

**FIGURE 1:**
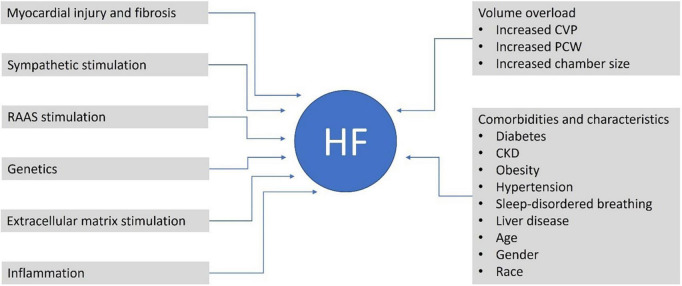
Potential contributors and mechanisms contributing to HF that could be altered by sacubitril/valsartan or are common characteristics of patients with HF. CKD, chronic kidney disease; CVP, central venous pressure; HF, heart failure; PCWP, pulmonary capillary wedge pressure; RAAS, renin-angiotensin-aldosterone system.

According to the US Guidelines, sacubitril/valsartan has a class 1 recommendation in HFrEF and 2b in HFmrEF and HFpEF, indicating the clinical benefits of treatment are most evident in patients with LVEF below normal (17). Potential mechanisms contributing to HF that could be altered by sacubitril/valsartan or contributing characteristics of patients who may experience HF are given in Figure 1.

Clinical trials have investigated the effects of sacubitril/valsartan compared with standard therapies in patients with HF (Table 2). Individual trials have generally evaluated populations within a specific LVEF range, making comparative and pooled data analyses challenging because of differences in trial patient selection criteria, control treatments, and endpoints.

**TABLE 2:** Key clinical trials investigating the effects of sacubitril/valsartan.

Study name and identifier	Design	Population	Primary endpoint and other key findings
**HFrEF**			
PARADIGM-HF (8) Dec 2009–May 2014 NCT01035255	Phase III, randomized, S/V vs. enalapril	*N* = 8442 LVEF ≤ 40%	Superiority of S/V vs. enalapril in reduction of the composite of CV death or HFH (21.8% vs. 26.5%; HR 0.80; 95% CI: 0.73–0.87; *P* < 0.001) over a median follow-up of 27 months
PIONEER-HF (39) April 2016–June 2018 NCT02554890	Phase III, randomized, S/V vs. enalapril	*N* = 881 LVEF ≤ 40% Patients hospitalized for ADHF	Greater time-averaged reduction in NT-proBNP with S/V vs. enalapril (ratio of change S/V vs. enalapril 0.71; 95% CI: 0.63–0.81; *P* < 0.001)
EVALUATE-HF Aug 2016–Dec 2018 NCT02874794	Randomized S/V vs. enalapril	*N* = 464 LVEF ≤ 40%	No significant difference in change in aortic characteristic impedance from baseline to week 12 between S/V and enalapril [−2.2 (95% CI: −17.6 to 13.2) dyne × s/cm^5^; *P* = 0.78]; S/V improved echocardiographic measurements vs. enalapril at week 12
PROVE-HF (29) Oct 2016–Oct 2018 NCT02887183	Phase IV Single arm Open-label	*N* = 794 LVEF ≤ 40%	At 12 months, the change in log_2_–NT-proBNP correlated with changes in: LVEF [r = −0.381 (IQR −0.448 to −0.310); *P* < 0.001] LVEDVI [r = 0.320 (IQR 0.246–0.391); *P* < 0.001] LVESVI [r = 0.405 (IQR 0.335–0.470); *P* < 0.001] LAVI [r = 0.263 (IQR 0.186–0.338); *P* < 0.001] E/e’ [r = 0.269 (IQR 0.182–0.353); *P* < 0.001]
LIFE (58) Mar 2017–Sept 2020 NCT02816736	Randomized, S/V vs. valsartan	*N* = 365 Advanced HF LVEF ≤ 35% NYHA class IV BNP ≥ 250 pg/ml or NT-proBNP ≥ 800 pg/ml SBP ≥ 90 mmHg ≥1 additional objective finding of advanced HF	No difference in the proportional change in the AUC for NT-proBNP levels from baseline to week 2, 4, 8, 12, and 24
**HFpEF**			
PARAMOUNT-HF (72) Nov 2009–Dec 2011 NCT00887588	Phase II, randomized, S/V vs. valsartan	*N* = 301 LVEF ≥ 45%	Greater NT-proBNP reduction at 12 weeks with S/V vs. valsartan (ratio of change 0.77; 95% CI: 0.64–0.92; *P* = 0.005); reduction in left atrial volume (*P* = 0.003) and dimension (*P* = 0.034) with S/V vs. valsartan at 36 weeks
PARAGON-HF (57) July 2014–June 2019 NCT01920711	Phase III, randomized, S/V vs. valsartan	*N* = 4822 LVEF ≥ 45%	No significant difference in composite of total* HFH and CV death (RR 0.87; 95% CI 0.75–1.01; *P* = 0.06); benefit with S/V in patients with LVEF ≤57% (RR 0.78; 95% CI 0.64–0.95)
PARALLAX (40) Aug 2017–Oct 2019 NCT03066804	Randomized, S/V vs. IMT	*N* = 2572 LVEF > 40%	Greater NT-proBNP reduction at 12 weeks with S/V [geometric mean ratio: 0.84 (95% CI 0.80–0.88; *P* < 0.001)] vs. IMT; no difference in 6MWD improvement
PERSPECTIVE Nov 2016–May 2022 NCT02884206	Phase III, randomized, S/V vs. valsartan	LVEF > 40% NYHA class II–IV NT-proBNP ≥ 125 pg/ml	Primary outcome: change from baseline in the Cogstate global cognitive composite score
PARAGLIDE-HF (Abstr: Ward J. Presented at HFSA 2021) June 2019–Oct 2022 (est.) (ongoing) NCT03988634	Phase III, randomized, S/V vs. valsartan	LVEF > 40% Patients with a WHF event (HFpEF decompensation) stabilized and initiated at the time of or within 30 days post-decompensation	Primary outcome: time-averaged proportional change in NT-proBNP from baseline to weeks 4 and 8
**Post-MI**			
PARADISE-MI Dec 2016–Feb 2021 NCT02924727	Phase III, randomized, S/V vs. ramipril	LVEF ≤ 40% Post-AMI	No significant difference in the primary outcome of death from CV causes or incident HF between S/V and ramipril (HR 0.90; 95% CI: 0.78–1.04; *P* = 0.17)

6MWD, 6 min walk distance; ADHF, acute decompensated heart failure; AMI, acute myocardial infarction; AUC, area under the curve; BNP, brain natriuretic peptide; CI, confidence interval; CV, cardiovascular; E/e’, early transmitral Doppler velocity/early diastolic annular velocity; HF, heart failure; HFH, hospitalization for heart failure; HFpEF, Heart failure with preserved ejection fraction; HFrEF, Heart failure with reduced ejection fraction; HR, hazard ratio; IMT, individualized medical therapy; IQR, interquartile range; LAVI, left atrial volume index; LV, left ventricular; LVEDVI, left ventricular end-diastolic volume index; LVEF, left ventricular ejection fraction; LVESVI, left ventricular end-systolic volume index; MI, myocardial infarction; NT-proBNP, N-terminal pro B-type natriuretic peptide; NYHA, New York Heart Association; RR, rate ratio; S/V, sacubitril/valsartan; SBP, systolic blood pressure; WHF, worsening heart failure.

*First and recurrent.

In addition to sacubitril/valsartan, sodium glucose co-transporter 2 (SGLT2) inhibitors are now included in guideline-directed medical therapy for HF. Similar to sacubitril/valsartan, they have a class 1 recommendation in HFrEF and 2a in HFmEF and HFpEF, based on their relative efficacy across the LVEF continuum. Moreover, they confer clinical benefits through complementary and non-overlapping mechanisms of action compared with sacubitril/valsartan. As a result, neither class is intended to be a replacement for, or interchangeable with, the other. Therefore, optimizing medical therapy with multiple medication classes may be an option for some patients with HF (17, 18).

## Molecular mechanisms of HF and markers of treatment response

### LVEF

**FIGURE 2:**
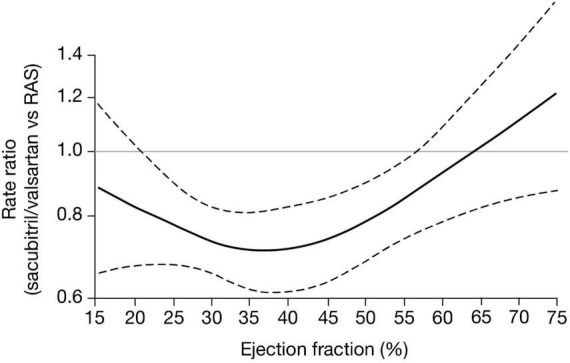
CI, confidence interval; RAS, renin-angiotensin-aldosterone-system inhibitor. Treatment effects of sacubitril/valsartan versus active comparator (either enalapril or valsartan) across a range of ejection fraction for the composite of total heart failure hospitalization and cardiovascular death. Estimated rate ratios and 95% CIs obtained from negative binomial regression models with ejection fraction expressed *via* restricted cubic spline (19).

The efficacy of sacubitril/valsartan varies with LVEF, with patients with ejection fractions below normal experiencing the greatest therapeutic benefits (17, 19). An analysis of pooled PARADIGM-HF (HFrEF) and PARAGON-HF (HFpEF) trial data (Figure 2) demonstrated multiple clinical benefits with sacubitril/valsartan compared with active control, including reduced HF hospitalization and cardiovascular-related mortality in patients with an LVEF of up to 60%. Benefits persisted to higher LVEF values in women than in men, with maximal benefit seen at an LVEF of ∼35%–40% (Figure 2; 19). Furthermore, in a PARAGON-HF secondary analysis that compared sacubitril/valsartan with valsartan alone, improvements with sacubitril/valsartan in the primary endpoints of total HF hospitalization and cardiovascular death extended to patients with an LVEF of up to 60% (20).

Although LVEF is a useful parameter, there are several limitations to using LVEF for HF categorization and guiding treatment decisions (15, 16). LVEF measurement can be imprecise, with low reproducibility, depending on the technique used and patient population. Echocardiography is most commonly used for LVEF assessment but also provides additional information about hemodynamic parameters (e.g., global longitudinal strain), which can assist in the evaluation of patients with HF taking sacubitril/valsartan (10, 21, 22); however, obesity and other conditions common in HFpEF may limit image quality. Moreover, echocardiography has significant interobserver variability. Other LVEF assessment techniques such as magnetic resonance imaging or computed tomography do not produce identical imaging and are generally less accessible (10). LVEF can also vary with HF treatment, blood pressure, heart rate, volume status, and rhythm, particularly with atrial fibrillation (16, 23, 24). Moreover, LVEF has a wide normal range and varies with patient sex, age, and ethnicity (6). Importantly, LVEF does not reflect underlying HF etiologies, and HF classification based exclusively on LVEF fails to represent the heterogeneity and complexity of this diagnosis (16). Use of additional criteria beyond LVEF for characterization may improve prediction of patient response to sacubitril/valsartan and other therapies.

### Cardiac remodeling and NPs

A central aspect of HF progression is cardiac remodeling, characterized by changes in wall thickness, mass, and cardiac chamber volumes (25; Figure 1).

Heart failure with reduced LVEF is usually accompanied by chamber enlargement with “eccentric” LV remodeling, which may be due to loss of cardiomyocytes and their replacement with fibrotic tissue and due to elongation of surviving myocytes. Most patients with chamber enlargement have diminished contractile function (26, 27). Conversely, HF with LVEF ≥40% is more often characterized by “concentric” LV remodeling with increased wall thickness and interstitial fibrosis in the context of a relative absence of myocyte loss, leading to LV stiffening and impaired LV filling and relaxation (1, 27).

In patients with HFrEF, treatment with guideline-directed medical therapy and cardiac resynchronization therapy often results in reverse cardiac remodeling and improved clinical outcomes (28). Sacubitril/valsartan improved measures of cardiac remodeling at 6 and 12 months in PROVE-HF (29) and improved echocardiographic measurements compared with enalapril at 12 weeks in EVALUATE-HF (30; Table 2). Furthermore, results from a meta-analysis showed that sacubitril/valsartan improved indices of cardiac reverse remodeling compared with angiotensin-converting enzyme inhibitors (ACEis)/angiotensin II receptor blockers (ARBs) in patients with HFrEF (31).

Neurohormonal imbalance, characterized by hyperactivation of the renin-angiotensin-aldosterone system and the sympathetic nervous system, and increased resistance to the NPs represent compensatory homeostatic responses to the decline in cardiac function in HF. Although beneficial in the short term, this neurohormonal imbalance promotes further myocardial damage and decompensation in the long term (32–34; Figure 2). NP concentrations are affected by LV size and volume and are associated with LV remodeling (35). In the PROVE-HF study, the concentrations of ANP doubled following initiation of sacubitril/valsartan in patients with HFrEF. Earlier and larger increases were associated with greater reverse cardiac remodeling, suggesting that changes in ANP might mediate the benefits of sacubitril/valsartan in chronic HFrEF (36).

Biomarkers indicative of cardiac strain or injury such as BNP and NT-proBNP are commonly used to diagnose HF and assess the current state of compensation. Both biomarkers are often elevated in patients with HF, with higher concentrations associated with increased cardiovascular risk in patients with HFrEF (5). However, BNP and NT-proBNP levels could also be affected by other patient factors including age, sex, body mass index, LVEF, and comorbidities (5). Although both biomarkers have diagnostic and prognostic value, NT-proBNP has proven superior in predicting HF mortality, HF morbidity, and hospitalization (5). Moreover, since NT-proBNP is not a substrate of neprilysin, its levels more accurately reflect the changes in myocardial wall stress following treatment with sacubitril/valsartan. In the PARADIGM-HF study, among patients with LVEF ≤40%, sacubitril/valsartan was superior to enalapril in reducing the composite risk of cardiovascular death and first HF hospitalization and the risk of death from any cause (8). Sacubitril/valsartan increased cGMP and plasma BNP levels, and lowered NT-proBNP and troponin levels, with significant differences first apparent within 4 weeks of initiation and sustained at 8 months. Among patients with NT-proBNP levels >1,000 mg/dl at baseline, NT-proBNP levels were reduced to ≤1,000 mg/dl at 1 month after randomization in 31% of those treated with sacubitril/valsartan versus 17% of those treated with enalapril (*P* < 0.001). The risk of HF hospitalization or cardiovascular mortality was 59% lower in patients who achieved NT-proBNP levels < 1,000 mg/dL than those who did not, with the relationship between changes in NT-proBNP and clinical outcomes independent of treatment group (37). In line with these findings, several studies have reported a significant effect of sacubitril/valsartan in reducing NT-proBNP levels (38–40). In a PARAGON-HF secondary analysis, reduced NT-proBNP levels were associated with lower risk of the primary endpoint, and treatment with sacubitril/valsartan reduced NT-proBNP compared with valsartan (41). Furthermore, in PROVE-HF, reduced NT-proBNP concentrations with sacubitril/valsartan treatment were associated with reverse cardiac remodeling (29; Table 2). Overall, these data suggest that NT-proBNP has prognostic value independent of HF therapy and NT-proBNP baseline levels.

Lower NP levels have been consistently reported for patients with preserved versus reduced LVEF, although the associated pathophysiologic implications are not fully understood. Low circulating NPs are associated with lower myocardial end-diastolic LV wall stress, which is anticipated in patients with normal or small ventricle size and normal or increased wall thickness, typical in those with LVEF in the normal range compared with those with LVEF ≤40%. Elevating NP levels in patients with LVEF in the normal range could potentially promote LV de-stiffening, cardiac unloading, and decongestion (42). However, a lower response to sacubitril/valsartan has been observed in patients with preserved versus reduced LVEF, suggesting that additional mechanisms may contribute to these differences (11).

Although NPs are established diagnostic and prognostic tools for HF, evidence supporting their effectiveness for treatment guidance is inconsistent (43). Furthermore, BNP levels are inversely correlated with body mass index, and patients with obesity may have BNP levels below the clinical threshold for HF (44). This is important since over half of the patients with HFpEF have obesity as a major comorbidity or driver of the syndrome.

### Additional biomarkers

Multiple factors including genetics, inflammation, altered metabolism, changes in cellular signaling, myocardial injury, and neurohormonal imbalance have been associated with changes in cardiac extracellular matrix. These changes include altered collagen synthesis and degradation, which can lead to increased collagen volume deposition and contribute to LV stiffening and chamber remodeling (32, 45–47).

By inhibiting both the angiotensin II receptor and neprilysin, sacubitril/valsartan has theoretical antifibrotic effects within the myocardium (1; Figure 2). This possibility is supported by the PIONEER-HF trial, in which biomarkers of myocardial injury (high-sensitivity troponin) and ventricular wall stress (soluble ST2 protein) were associated with HF patient hospitalization and cardiovascular death. Both of these biomarkers were reduced by sacubitril/valsartan in patients with acute decompensated HF (48). In addition, in a PARAGON-HF biomarker sub-study including patients with HF and an LVEF of ≥45%, the population had elevated levels of circulating biomarkers of extracellular matrix homeostasis. Higher soluble ST2 and tissue inhibitor of matrix metalloproteinase one levels at baseline and increases at week 16 were significantly associated with greater risks for cardiovascular death and HF hospitalization. Treatment with sacubitril/valsartan significantly altered extracellular matrix biomarkers levels compared with valsartan alone, suggesting favorable changes in extracellular matrix homeostasis (38). These results suggest that antifibrotic properties may contribute to the benefit of sacubitril/valsartan in patients with HF.

In a network analysis of the PROTECT trial, biomarker profiles differed between patients with HFrEF (LVEF < 40%), HFpEF (LVEF ≥ 50%), and HFmrEF (LVEF 40–49%). Interactions between biomarkers were mainly related to cardiac stretch in HFrEF and inflammation in HFpEF, with an intermediate biomarker profile for patients with HFmrEF (49). Inflammation may be a major factor contributing to HFpEF, and treatments that reduce inflammation, including sacubitril/valsartan, may be beneficial in this patient population.

### Comorbidities and burden of HF

Comorbidities such as hypertension, kidney disease, and diabetes are potential causative factors in HF development that may increase symptom burden, contribute to HF progression, and significantly affect treatment options (50, 51). Beyond its effect on the renin-angiotensin-aldosterone system, sacubitril/valsartan increases NP and bradykinin activity, reducing systolic blood pressure more than ACEis and ARBs (52, 53).

Some HF therapies can aggravate renal impairment (54). In a secondary analysis of PARADIGM-HF, compared with enalapril, sacubitril/valsartan had beneficial effects on renal and cardiovascular outcomes that were consistent in patients with or without chronic kidney disease. Moreover, the decrease in estimated glomerular filtration rate from baseline to 12 months was smaller in patients receiving sacubitril/valsartan versus enalapril. Although a small increase in urinary albumin-to-creatinine ratio was observed, it was not associated with cardiovascular outcomes (55). Similar findings were observed in PARAGON-HF, where sacubitril/valsartan treatment of patients with HFpEF was associated with a slower decline in estimated glomerular filtration rate and a reduced risk of the prespecified renal composite outcome by 50% versus valsartan (56).

Patients with HF often develop hyperkalemia owing to comorbidities such as renal impairment and diabetes mellitus or the use of mineralocorticoid antagonist therapy; therefore, regular monitoring of serum potassium is recommended. Compared with valsartan (PARAGON-HF) or enalapril (PARADIGM-HF), treatment with sacubitril/valsartan resulted in less frequent hyperkalemic adverse events (8, 57). However, among patients with advanced HF, non–life-threatening hyperkalemia occurred more frequently with sacubitril/valsartan versus valsartan (17 vs. 9%; *P* = 0.04) (58). Severe hyperkalemia was less likely among patients on mineralocorticoid receptor antagonists with sacubitril/valsartan versus enalapril, suggesting that sacubitril/valsartan may increase the tolerability of mineralocorticoid receptor antagonists compared with ACEis (59).

Approximately 30–50% of patients with HF have type 2 diabetes mellitus (60–63). In a 3 years follow-up post-hoc analysis of PARADIGM-HF, patients with diabetes and HFrEF receiving sacubitril/valsartan had a greater reduction in hemoglobin A1c than those on enalapril. Furthermore, initiation of insulin was lower with sacubitril/valsartan than with enalapril. This improved glycemic control with sacubitril/valsartan could be due to neprilysin inhibition and subsequent increases in NP, bradykinin, and cGMP pathway levels, potentially influencing insulin sensitivity and metabolism (64). A post-hoc analysis of the PROVE-HF study showed that sacubitril/valsartan favorably impacted change from baseline to 12 months in LVEF, Kansas City Cardiomyopathy Questionnaire (KCCQ-23) overall summary scores, and NT-proBNP levels, regardless of diabetes status (65). Similarly, an analysis of PARADIGM-HF showed benefits on cardiovascular mortality or HF hospitalization with sacubitril/valsartan irrespective of diabetes status (66).

### Treatment response in different patient populations

Differences in response to sacubitril/valsartan treatment may exist between men and women and different races and ethnicities. In PARAGON-HF, among patients with ejection fraction ≥45%, compared with valsartan, sacubitril/valsartan reduced the risk of HF hospitalization more in women than in men (67). Sex-based differences were also observed in a PROVE-HF subgroup analysis in patients with ejection fraction ≤40% treated with sacubitril/valsartan. Compared with men, women showed a more rapid early decrease in NT-proBNP after initiation of sacubitril/valsartan. In addition, women had worse baseline KCCQ-23 Total Symptom scores than men but showed greater early improvement after initiating sacubitril/valsartan. Both men and women showed similar degrees of reverse cardiac remodeling after initiating sacubitril/valsartan; however, these changes occurred earlier in women (68). Similarly, changes in NT-proBNP, cardiac reverse remodeling and health status scores were generally similar in Black, Hispanic, and White patients with HFrEF, although the patterns of changes were subtly different between groups (69). It has been shown that HF is more prevalent and associated with higher mortality and morbidity among Black patients than among White patients. Moreover, differences in response to treatment with an ACEi have been reported between Black patients and patients of other races (70). Conversely, prespecified analysis of PIONEER-HF that compared the effect of in-hospital initiation of sacubitril/valsartan and enalapril in patients with acute decompensated HFrEF showed a greater reduction in the composite endpoint of HF rehospitalization or cardiovascular death with sacubitril/valsartan, with no significant differences observed between Black and non-Black patients (71).

## Conclusion

Left ventricular ejection fraction has been historically used as the main factor in HF categorization and for guiding treatment choices. Although clearly useful, this approach has inherent limitations. Recent studies have demonstrated that the benefits of sacubitril/valsartan in the treatment of HF may extend across the spectrum of LVEF, with benefits most evident in patients with LVEF below normal. Consideration of other disease features may help to guide the choice of treatments since patterns of cardiac remodeling, neurohormonal imbalance, and changes in cardiac extracellular matrix and type of cardiac dysfunction occur across the continuum of HF. Patterns of cardiac remodeling, biomarkers such as NT-proBNP reflecting fibrosis and inflammation, as well as comorbidities, patient characteristics, and signs and symptoms should be investigated to define patient subgroups, with greater potential for a better response to mechanism-based therapies, including sacubitril/valsartan.

## Author contributions

CE and SL: conceptualization, methodology, writing—original draft, critical revision, and final approval for submission. Both authors contributed to the article and approved the submitted version.
